# Knowledge, Attitude, and Practices About Research Integrity and Scientific Misconduct Among the Faculty and Medical Postgraduates Working in Medical Colleges in North Karnataka and Central India: A Cross-Sectional Online Survey

**DOI:** 10.7759/cureus.59200

**Published:** 2024-04-28

**Authors:** Anant Khot, Chaitali A Chindhalore, Akram Naikwadi

**Affiliations:** 1 Pharmacology, All India Institute of Medical Sciences, Nagpur, IND; 2 Pharmacology and Therapeutics, Bijapur Lingayat District Educational (BLDE) Shri B. M. Patil Hospital, Medical College and Research Center, Vijayapura, IND

**Keywords:** survey, research integrity, questionable research practices, scientific misconduct, cross-sectional studies

## Abstract

Introduction

Research integrity is an active adherence to the ethical principles and professional standards essential for the responsible practice of research. Research or scientific misconduct stands like child abuse today. The survey of National Institutes of Health (NIH)-funded scientists calculated an absolute minimum of 2325 incidents of scientific misconduct per year. A report has also shown that Iran (6.60), India (5.68), Turkey (5.38), South Korea (3.59), and China (2.00) had higher ratios of publication misconduct to distrust data or interpretations than other countries. Hence, to determine the knowledge, attitude, and practices (KAPs) of the research integrity/scientific misconduct among the faculty and postgraduates working in the medical colleges in North Karnataka (NK) and Central India (CI), this study has been carried out.

Methods

It is a web-based, cross-sectional study carried out with the use of Google Forms (Google, Mountain View, California). A pretested, unstructured questionnaire consisting of 25 questions was posted in the way of a link to the faculty and postgraduates working in various disciplines within the colleges of NK and CI either by using an e-mail or other social platforms like WhatsApp. Institutional Ethics Committee approval was obtained in both regions before conducting the survey.

Results

A total of 146 participants responded to the e-questionnaire posted to them. Participants from CI displayed better awareness in several areas compared to NK. Citing articles and/ or materials that have not been read is the common questionable research practice (QRP) they have come across, as mentioned by participants in both groups.

Discussion

The study reveals a moderate level of knowledge and variable attitudes toward research integrity. The "publish or perish" culture is a major contributor to misconduct. Training and awareness programs are needed to enhance ethical research practices.

Conclusion

This study highlights the need for improved education and policy implementation to uphold research integrity in medical colleges, emphasizing the role of academic culture in shaping ethical research practices.

## Introduction

Research integrity is an active adherence to the ethical principles and professional standards essential for responsible research practice [1]. Scientists involved in research shall possess specific shared values like honesty, accuracy, efficiency, and objectivity [2]. Also, they should comply with ethical standards set by different agencies, such as the Indian Council of Medical Research (ICMR). Society's confidence in and support of research largely rests on public trust in the integrities of individual researchers and their supporting institutions [3]. However, the scenario is changing; maybe due to hyper-competition among the faculty/researchers and the enforcement by the regulatory bodies, the scientists are engaged in unethical practices or scientific misconduct [4]. The US Department of Health and Human Services Office of Research Integrity (ORI) defines misconduct as "fabrication, falsification, or plagiarism (FFP) in proposing, performing, or reviewing the research, or in reporting research results" [5]. Misconduct does not include honest error or disagreement about methods, interpretations of data, or scientific issues [6].

The recent systematic review of studies shows frighteningly high levels of misconduct in high-income countries. The survey of National Institutes of Health (NIH)-funded scientists calculated an absolute minimum of 2325 incidents of scientific misconduct per year [7]. A report has also shown that Iran (6.60), India (5.68), Turkey (5.38), South Korea (3.59), and China (2.00) had higher ratios of publication misconduct to distrust data or interpretations than other countries [8].

Research misconduct is now acknowledged as a significant issue; it was overlooked in the past, and we are witnessing its prevalence more frequently today. An analysis posted on the Nature Journal's blog found that India had the highest fraud rate in the world - 18 papers out of 100,000 [9]. This may be due to factors like the "publish or perish" culture in academia, inadequate training, investigator laziness, and insufficient monitoring [10]. A trend of "hyper authorship" or "mass authorship", writing articles with more than 1000 co-authors, has increased about two-fold over five years. To promote research integrity at Higher Educational Institutions (HEIs), the University Grants Commission (UGC) laid down regulations in the year 2018 and also published a guidance document on Good Academic Research Practices (GARP) in 2020 [11, 12]. ICMR bioethics unit has also published a policy on Research Integrity and Publication Ethics in 2019. Despite all the measures, breaches of research integrity, especially detrimental authorship practices or questionable research practices, are widely prevalent [13]. Hence, this study has been carried out to determine the knowledge, attitude, and practices of research integrity/scientific misconduct among the faculty and postgraduates working in the medical colleges in North Karnataka and Central India.

## Materials and methods

This study is a web-based, cross-sectional study that was carried out using Google Forms. A pretested, unstructured questionnaire consisting of 25 questions was posted as a link to the faculty and postgraduates working in various disciplines within the 13 colleges of North Karnataka (NK) and 15 medical colleges in Central India (CI) covering Vidarbha region of Maharashtra, Madhya Pradesh, and Chhattisgarh either by using e-mail or other social platforms like WhatsApp. These two zones are selected due to a job change by the principal investigator from NK to CI. Informed consent was obtained from all the participants through the Google Form (Google, Mountain View, California), and an Institutional Ethical Clearance was obtained before starting the study in both centers. Though the initial research was planned for one month, it was extended because of the inadequate response of the participants. The study was planned to be published separately in two zones, but the results are combined and compared to avoid duplicate publication. Responses obtained were exported to the Microsoft Excel spreadsheet. Both Microsoft Excel 365 (Microsoft, Redmond, Washington) and Statology two-proportion Z test calculator were used for data representation and statistical analysis. The two-proportion Z test was applied to compare the response between both groups.

## Results

A total of 146 participants responded to the e-questionnaire posted to around 300 people. Seventy-eight participants were from North Karnataka, and 68 were from medical colleges in central India. Table 1 mentions the demographic characteristics of participants from both groups. Both groups are comparable in demographic characteristics.

**Table 1:** Comparison of demographic characteristics of participants in both regions *Chi-square test is used

Parameter	North Karnataka (n=78)	Central India (n=68)	p-value*
Gender			
Male	41 (52.5%)	39 (57.3%)	0.37
Female	35 (44.8%)	29 (42.6%)	
Not specified	2 (2.5%)	0	
Cadre			
Professor	12 (15.3%)	10 (14.7%)	0.88
Associate professor	19 (24.3%)	16 (23.5%)	
Assistant professor	20 (25.6%)	22 (32.3%)	
Senior resident	5 (6.4%)	6 (8.8%)	
Postgraduate	20 (25.6%)	13 (19.1%)	
Not specified	2 (2.5%)	1 (1.4%)	

The responses to the knowledge questions by both groups were compared, and the results are depicted in Table 2

**Table 2:** Comparison of the responses to knowledge questions in both regions * Two-proportion Z test is used and p <0.05 is considered statistically significant ICMJE - International Committee of Medical Journal Editors

Questions and correct responses after the question	North Karnataka (n= 78)	Central India (n=68)	p-value
Are you aware of the Research Integrity and Publication Ethics (RIPE) policy? Yes	46 (58.9%)	38 (55.8%)	0.71
The Office of Research Integrity (ORI) definition of research misconduct includes all the following, Except? Detrimental authorship practices	35 (44.8%)	41(60.2%)	0.05*
If you use undergraduate students or other residents as research subjects without informing the overseeing Dean, program director, or other pertinent officials, which ethical principle of biomedical research will you be violating? Principle of Justice	5 (6.41%)	3 (4.41%)	0.58
Author X has submitted a systematic review article to a National Journal. A peer reviewer has commented that the parts of this paper have been reproduced from the work previously published by the same author, this is referred to as? Self-plagiarism	36 (46.1%)	43 (63.2%)	0.03*
Similarity checks for plagiarism shall exclude all the following, except? Common knowledge or coincidental terms of more than 14 consecutive words	32 (41%)	30 (44.1%)	0.71
If your colleague has the ambition to reach a higher academic position quickly, he tried to plagiarize with a similarity index of 52%. For which, his article was withdrawn, was deprived of an annual increment & he was told by the Dean, that he should not guide any postgraduate for the next two years. Considering the quantum of his punishment, what is the level of plagiarism he would have executed? Level 2	22 (32.3%)	37 (54.4%)	0.0007*
Copying phrases, passages, and ideas from different sources and putting them together to create a new text is termed as? Mosaic plagiarism	18 (23%)	28 (41.17%)	0.01*
If you combine correctly cited sources with those copied without citation, then it is referred to as? Hybrid	38 (48.7%)	22 (32.3%)	0.04*
A senior academician or a pioneer in your research interest area who does not meet the ICMJE criteria that would qualify him/her as an author. But you want to mention his/her name in your article to indicate his/her reference in the future. In such a scenario, he/she will be called as? Guest author	35 (44.8%)	32 (47%)	0.71
How do you rate the effectiveness of your organization's rules and procedures for reducing scientific misconduct?			
High	29 (37.1%)	21 (30.8%)	0.37
Average	31 (39.7%)	24 (35.2%)	0.61
In all the following conditions, informed consent is not necessary or will not be considered as scientific misconduct, except? Research involving an incompetent individual	23 (29.4%)	17 (25%)	0.58

As per Table 2, the faculty and postgraduates in CI (60.2%) were better informed than NK (44.8%) about the ORI definition of research integrity. Similarly, the knowledge about self-plagiarism, mosaic plagiarism, and level of plagiarism was significantly better among faculty and medical postgraduates working in CI. Both the zones rated the effectiveness of their organization’s rules and procedures as high to the average for reducing scientific misconduct. More respondents from the NK answered correctly only for the question on the hybrid type of plagiarism. The remaining responses to questions for the knowledge assessment were mostly comparable, and few were asked only in one region because of the modifications suggested by the research cell in the latter region. For the questions asked only in the NK region covered the knowledge about Good Laboratory Practice (GLP), 39.7% responded correctly; for International Committee of Medical Journal Editors (ICMJE) authorship criteria, 50% responded correctly; and for guidelines followed for biomedical research in their zone, 73% participants mentioned about ICMR guidelines. The questions in CI covered the Institutional Academic Integrity Panel (IAIP) knowledge. Only 7.3% of the participants knew IAIP in their institution. The attitude question responses are summarized in Table 3.

**Table 3:** Comparison of the responses to attitude questions in both regions * Two-proportion Z test is used and p<0.05 is considered statistically significant CME - continuing medical education; GCP - good clinical practice; IEC - Institutional Ethics Committee; PI - principal investigator

Questions	North Karnataka, n= 78	Central India, n=68	p-value
Do you feel that failure to disclose the relevant financial or intellectual conflicts of interest should be considered as "Research misconduct"?	Yes, 48 (61.5%)	Yes, 53 (77.9%)	0.03**
Which among the following Questionable Research Practices (QRPs) have you come across?			
Carrying out the study without the approval from an Institutional ethics committee	15	11	
p-hacking	8	1	
Salami slicing	8	11	
Citing articles and/ or materials that have not been read	24	23	
None of the above	20	10	
Not answered	11	10	
Photomanipulation	0	4	
Plagiarism and duplication of research work	0	27	
Keeping names of seniors as authors without their active role in research work	0	15	
Adding names of spouses, and friends as authors in an article without the significant contribution from them to the study	0	11	
Do you think that the "Whistleblower's Bill" needs to be made mandatory for all Academic Institutions to expose the Politics of Publication?	Yes, 38 (48.7%)	Yes, 41 (60.2%)	0.14
Which of the following reasons do you think are responsible for or promote the researchers to indulge in detrimental/QRPs?			
Publish or perish attitude of administrators/Academic competitiveness	20	14	
An unrealistic demand for perfect results	7	4	
Poorly trained students/researchers	9	2	
Duplication, salami slicing, and gift authorship reap in great rewards	7	2	
Lack of value-based education in the early education curriculum	7	1	
Not answered	0	5	
Do you feel that the "balloon professors" exist in your organization and need to evaluate their contribution to the article they have authored or those claiming to have so many publications? Yes	33 (42.3%)	25 (36.7%)	0.45
The following questions are unique to the Central India			
Responsibility for the scientific integrity of a study/project lies with			
Principal investigator	60		
Ethics committee	21		
Sponsor/funding agency	12		
Head of the Department	6		
Not answered	4		
What do you think a professional colleague in your work environment would do if they knew a Principal Investigator or co-investigator had violated the accepted norms of research integrity on a project?			
Express the disapproval in private but will not report	24 (35.2%)		
Ask the investigator to report by him/herself, and report if they fail to do so	15 (22%)		
Probably nothing	16 (23.5%)		
Report to the appropriate authorities	9 (13.2%)		
Not answered	4 (5.8%)		
According to you what measures need to be adopted to prevent scientific misconduct or to protect the integrity of research			
Enough time to be given to study research methodology from the beginning of medical school			
Mandatory registration of every thesis, project work, or dissertation on the Clinical Trial network			
Strict implementation by the IEC, Proper GCP training of Investigators, students, and IEC members, free available plagiarism checking software by the university to dissertation assessors, and equal weightage to all authors			
CMEs/ workshops should be organized			
At the department level, postgraduate students should also be made aware of the subject above-mentioned			
The whistleblower bill should be legalized			
Raising awareness of best practices in the Responsible Conduct of Research			
Train from early formative years on proper research practices			
Pressure for publication should be stopped. More training needs to be taken.			
Value education early in the career			
Taking more awareness workshop			
The environment needs to be more supportive and accommodating for everyone			
Research should be a choice, not a compulsion.			

According to Table 3, more respondents in CI feel that if conflicts of interest are not disclosed, they need to be considered as research misconduct. 60.2% of respondents from medical colleges in CI feel that the Whistleblower's Bill is made mandatory to avoid politics of publication.

The respondents in the NK region mentioned citing articles and/ or materials that have not been read as the prominent Questionable Research Practice (QRP), which they came across, and other QRPs reported in both groups are mentioned in Table 3. Sixty of respondents in CI feel that the responsibility for the scientific integrity of a study lies with the principal investigator. Publish or perish attitude of administrators/Academic hyper-competitiveness is the primary reason cited by the participants, which encourages researchers to indulge in QRPs in both regions. The participants felt that proper training early in the formative years and reducing the pressure to publish the QRP’s can be prevented. The practice questions and their responses are summarized in Table 4. 

**Table 4:** Comparison of the practice questions from both regions *Two-proportion Z test is used and p <0.05 is considered statistically significant

Questions	NK (n= 78)	CI (n=68)	p-value
Which software do you use for similarity check or plagiarism check at your Institution?			
Turnitin	12 (15.3%)	9 (13.2%)	
Urkund	34 (43.5%)	7 (10.2%)	
iThenticate	4 (5.1%)	9 (13.2%)	
Cross-ref	6 (7.6%)	2 (2.9%)	
Grammarly	0	8 (11.7%)	
Plagcheck	0	8 (11.7%)	
None of the above	11 (14.1%)	19 (27.9%)	
Not answered	11 (14.1%)	08 (11.7%)	
Have you anytime published an article prematurely and in a predatory journal to disseminate your results quickly, with or without pressure from sponsors/academic pressure	Yes, 18 (23%)	Yes, 5 (7.3%)	0.007*
Have you witnessed any of the following entities/ unethical research practices in your workplace and felt the need to report to the officials of the College/University			
Intentional protocol violations related to subject enrolment or procedures	14	3	
Selective dropping of data from ‘outliers’	18	2	
Pressure from study sponsor to engage in unethical practices	7	4	
Collusion	5	2	
Not answered	29	12	
No/None	0	39	
What consequences you have heard or witnessed due to lack of research integrity in your organization?			
Harm to individuals and society	10	14	
Creating false leads for other scientists to follow- Direct damage to science	21	35	
The degradation of relations among scientists/faculty with their colleagues	20	20	
Damage to the public trust in science/an organization	15	27	
Waste of time, effort & money to reproduce fraudulent results	32	28	
Dishonesty & misrepresentation of data is common in society and does not hurt anybody	0	8	
Question asked only in Central India			
In your work environment, how often do you find that the investigators are engaged in scientific misconduct in the last 1-2 years?			
Never	34 (50%)		
Once	11 (16.1%)		
2-5 times	16 (23.5%)		
6-10 times	01 (1.4%)		

It has predominantly descriptive data mentioning the practices to maintain the research integrity like use software’s to check for similarity. Also, the premature and publication within predatory journals is less common for both the groups.

## Discussion

In this study, we assessed the knowledge, attitudes, and practices about research integrity/ scientific misconduct in two regions of India. The faculty and postgraduates working in medical colleges in Central India fared well in knowledge questions, except for the "hybrid" plagiarism question. Only 7.35% of respondents in CI knew about the Institutional Academic Integrity Panel, and only 50% of respondents in NK knew about ICMJE authorship criteria. The "publish or perish" attitude of administrators/ academic hyper-competitiveness is the primary reason cited by the participants, which promotes researchers to indulge in QRPs in both regions. This is similar to the reason quoted in an article by Mousavi et al. [14]. The participants feel that failure to disclose the relevant financial or intellectual conflicts of interest should be considered "research misconduct." Also, they responded that the "Whistleblower's Bill" needs to be made mandatory for all academic institutions to expose the Politics of Publication. Few participants accepted that they had published an article prematurely and in a predatory journal. Also, they have witnessed intentional protocol violations related to subject enrolment and selective dropping of data (outliers) at their workplace. In CI, 41.1% of respondents mentioned that the investigators in their work environment have engaged in scientific misconduct with varied frequency in the last one to two years. The results are similar to the study conducted by Kaiser et al. [15] and Ljubenkovi'c et al. [16]. However, they have estimated the prevalence of self-reported FFPs and QRPs separately, and we have not done the same to reduce the number of non-responders. If you ask a straight question like how many times you have falsified or fabricated the data, they will not fill out the Google form. 

However, with increased awareness about plagiarism, people have started to use one or the other software to check for plagiarism. Also, they know about the adverse consequences of a lack of research integrity within the organization. However, the problem of authorship and other QRPs are widely prevalent in both setups. The faculty usually exploits postgraduates and senior residents for personal gain. Also, the postgraduates and senior residents are unaware of QRPs, including authorship issues. They will commit scientific misconduct to fulfill the criteria for completing a degree and getting a job in a reputed medical college. A breach in the integrity of the research can be reduced by conducting CME and workshops on the responsible conduct of research. Further research in this direction is needed to reduce the menace of scientific misconduct. 

Limitations of the study

Few of the respondents felt the questions were difficult for them, and the answers could have been rated using a Likert scale. So, that is the maximum response we could have gotten. Non-responders have undermined the quality of the study. Considering the design, the generalizability of the study findings is limited.

## Conclusions

According to this study, though the knowledge is average in both regions, the respondents in the CI fared well in some of the knowledge questions asked. Attitude toward research integrity and scientific misconduct is variable due to academic hyper-competitiveness. They use different software to check for plagiarism. However, practices such as protocol deviation and selective dropping of the data (outliers) are prevalent though at lesser frequency, and training in the form of CME and workshops with a focus on responsible conduct of research can reduce the QRPs.

## Appendices

Study questionnaire:

1. Are you aware of the Research Integrity & Publication Ethics (RIPE) policy?

·  Yes

·  No

2. ORI's definition of Research misconduct includes all of the following, except?

a. Fabrication

b. Detrimental authorship practices

c. Falsification

d. Plagiarism

3. In your work environment, how often do you find that the investigators are engaged in scientific misconduct in the last 1-2 years?

a. Never

b. Once

c. 2-5 times

d. 6-10 times

e. > 10 times

4. Suppose you use undergraduate students or other residents as research subjects without informing the Dean, program director, or other pertinent officials. You will be violating which ethical principle of biomedical research?

a. Respect for autonomy

b. Beneficence

c. Non-maleficence

d. Justice

5. Author X has submitted a systematic review article to a National Journal. A peer reviewer has commented that the parts of this paper have been reproduced from the work previously published by the same author; this is referred to as

a. Self-plagiarism

b. Obfuscation

c. Fabrication

d. Duplicate publication

6. Which software do you use for similarity check or plagiarism check at your Institution?

a. Crossref

b. Turnitin

c. iThenticate

d. Urkund

e. Not so often used of late

f.  Plagcheck

g. Grammarly

7. Similarity checks for plagiarism shall exclude all of the following, except?

a. All quoted work reproduced with all necessary permission and/or attribution

b. All references and acknowledgments

c. All generic terms, laws, standard symbols & equations

d. Common knowledge or coincidental terms of more than 14 consecutive words

8. If your colleague has the ambition to quickly reach a higher academic position, he tried to plagiarise with a similarity index of 52%. His article was withdrawn, was deprived of an annual increment & he was told by the Dean, that he should not guide any postgraduate for the next two years. Considering the quantum of his punishment, what is the level of plagiarism he would have executed?

a. Level 0

b. Level 1

c. Level 2

d. Level 3

9. A senior academician or a 𝒑𝒊𝒐𝒏𝒆𝒆𝒓 in the area of your research interest who does not meet the ICMJE criteria that would qualify him/her as an author. But you want to mention his/her name in your article to indicate his/her reference in the future. In such a scenario, he/she will be called as

a. Ghost author

b. Guest author

c. Rolling author

d. Coercive author

10. Which among the following Questionable Research Practices (QRPs), you have come across in your immediate work environment? (Multiple responses)

o  Citing articles and/ or materials that they have not read

o  p-hacking

o  Salami slicing

o  Carrying out the study without the approval from an Institutional ethics committee

o  Photomanipulation

o  Plagiarism and duplication of research work

o  Keeping names of seniors without their active role in research work

o  Adding names of spouses, and friends as author

11. Have you anytime published an article prematurely and in a predatory journal to disseminate your results quickly, with or without pressure from sponsors/academic pressure

a. Yes

b. No

c. Maybe

12. Do you feel that the "𝗕𝗮𝗹𝗹𝗼𝗼𝗻 𝗣𝗿𝗼𝗳𝗲𝘀𝘀𝗼𝗿𝘀" exist in your organization and need to evaluate their contribution in the article they have authored or those claiming to have so many publications

a. Yes

b. No

c. Maybe

13. Do you feel that failure to disclose the relevant financial or intellectual conflicts of interest should be considered as “Research misconduct”?

a. Yes

b. No

c. Maybe

14. Responsibility for the scientific integrity of a study/project lies with

a. Principal Investigator

b. Sponsor/funding agency

c. Ethics committee

d. Head of the Department

e. Other …………..

15. Which of the following reasons do you think are responsible for or promote the researchers to indulge in detrimental/Questionable research practices?

a. Publish or perish attitude of administrators/Academic competitiveness

b. An unrealistic demand for perfect results

c. Poorly trained students/researchers

d. Duplication, salami slicing, and gift authorship reap in great rewards

e. Lack of value-based education in the early education curriculum

16. What consequences you have heard or witnessed due to the lack of research integrity in your organization?

a. Harm to individuals and society

b. Creating false leads for other scientists to follow- Direct damage to science

c. The degradation of relations among scientists/faculty with their colleagues

d. Damage to the public trust in science/an organization

e. Waste of time, effort & money to reproduce fraudulent results

f.  Dishonesty & misrepresentation of data are common in society and don't hurt anybody

17. How do you rate the effectiveness of your organization's rules and procedures for reducing scientific misconduct?

a. Very low

b. Low

c. Average

d. High

e. Very high

18. Does your college/organization have an Institutional Academic Integrity Panel (IAIP)?

a. Yes

b. No

c. Maybe

19. Informed consent is not necessary for all of the following conditions 𝐨𝐫 will not be considered as scientific misconduct, Except?

a. Research during humanitarian emergencies, military operations & disasters

b. Research on anonymized biological samples/data

c. Research on data available in the public domain

d. Research involving an incompetent individual

20. Copying phrases, passages, and ideas from different sources and putting them together to create a new text is termed as

a. Verbatim plagiarism

b. Accidental Plagiarism

c. Mosaic plagiarism

d. Paraphrasing

21. If you combine correctly cited sources with those copied without citation, then it is referred to as

a. CLONE

b. EMIX

c. HYBRID

d. MASH UP

22. Have you witnessed any of the following entities/ unethical research practices in your workplace and felt the need to report to the officials of the College/University?

a. Intentional protocol violations related to subject enrolment or procedures

b. Selective dropping of data from ‘outlier’ cases

c. Pressure from study sponsor to engage in unethical practices

d. Collusion

e. Other:……………………………………….

23. Do you think that the “Whistle-blower’s bill” needs to be made mandatory for all academic institutions to expose the Politics of Publication?

a. Yes

b. No

c. Maybe

24. What do you think a professional colleague in your work environment would do if they knew a Principal Investigator or co-investigator had violated the accepted norms of research integrity on a project?

a. Probably nothing

b. Express the disapproval in private, but will not report

c. Ask the investigator to report by him/herself, report if they fail to do so

d. Report to the appropriate authorities

25.  According to you what measures need to be adopted to prevent scientific misconduct or to protect the integrity of research.

Open-ended question:………
